# *In Vivo* Detection of Amyloid Plaques by Gadolinium-Stained MRI Can Be Used to Demonstrate the Efficacy of an Anti-amyloid Immunotherapy

**DOI:** 10.3389/fnagi.2016.00055

**Published:** 2016-03-22

**Authors:** Mathieu D. Santin, Michel E. Vandenberghe, Anne-Sophie Herard, Laurent Pradier, Caroline Cohen, Thomas Debeir, Thierry Delzescaux, Thomas Rooney, Marc Dhenain

**Affiliations:** ^1^Centre National de la Recherche Scientifique, Université Paris-Sud, Université Paris-Saclay, UMR 9199, Neurodegenerative Diseases LaboratoryFontenay-aux-Roses, France; ^2^Commissariat à l’Energie Atomique et aux Energies Alternatives, Direction de la Recherche Fondamentale, Institut d’Imagerie Biomédicale, MIRCenFontenay-aux-Roses, France; ^3^Sanofi, Neurodegeneration and Pain UnitChilly-Mazarin, France; ^4^Sanofi, Chilly-MazarinFrance

**Keywords:** Alzheimer, amyloid, gadolinium, immunotherapy, MRI

## Abstract

Extracellular deposition of β amyloid plaques is an early event associated to Alzheimer’s disease. Here, we have used *in vivo* gadolinium-stained high resolution (29^∗^29^∗^117 μm^3^) magnetic resonance imaging (MRI) to follow-up in a longitudinal way individual amyloid plaques in APP/PS1 mice and evaluate the efficacy of a new immunotherapy (SAR255952) directed against protofibrillar and fibrillary forms of Aβ. APP/PS1 mice were treated for 5 months between the age of 3.5 and 8.5 months. SAR255952 reduced amyloid load in 8.5-months-old animals, but not in 5.5-months animals compared to mice treated with a control antibody (DM4). Histological evaluation confirmed the reduction of amyloid load and revealed a lower density of amyloid plaques in 8.5-months SAR255952-treated animals. The longitudinal follow-up of individual amyloid plaques by MRI revealed that plaques that were visible at 5.5 months were still visible at 8.5 months in both SAR255952 and DM4-treated mice. This suggests that the amyloid load reduction induced by SAR255952 is related to a slowing down in the formation of new plaques rather than to the clearance of already formed plaques.

## Introduction

Alzheimer’s disease (AD) is the most common neurodegenerative disease of the central nervous system. It is characterized by two major microscopic lesions: β amyloid deposits in the form of extracellular amyloid β (Aβ) plaques and neurofibrillary tangles (NFTs) made of abnormal intracellular tau protein aggregates (Braak and Del Tredici, 2015). Aβ is constantly produced in the human brain where it normally remains in a soluble state (Haass and Selkoe, 2007; Eisele, 2013). However, Aβ peptides can adopt alternative conformations that aggregate into oligomeric, then proto-fibrillar and finally fibrillar forms that constitute the amyloid plaques (Eisele, 2013). Oligomeric Aβ induces synaptic toxicity (Lacor et al., 2004), while amyloid plaques lead to neuroinflammation (Serpente et al., 2014) and are also a reservoir for toxic soluble forms of Aβ (Craft et al., 2002). Limiting the amyloid load in the brain is therefore a potential therapeutic strategy for AD. Active and passive Aβ immunotherapy is the principle that has come furthest, both in number and in stage of clinical trials. These therapies can be efficient via a large number of mechanisms of action (Fu et al., 2010; Wisniewski and Goni, 2015). However, major difficulties have been reported in identifying clinical benefits in clinical trials initiated in symptomatic phases of the disease. They could be explained by the late initiation of the trials and a consensus is that future trials need to be performed in very early stages of the disease. This strategy is supported by recent data suggesting that a regionally limited tauopathy precedes Aβ pathology and in order to spread to the whole brain, the tau pathology requires the concomitant presence of Aβ pathology (Delacourte et al., 2002; Jack et al., 2013; Braak and Del Tredici, 2015). Refinements of anti-amyloid immunotherapies are ongoing to develop new therapies with mechanisms of action that improve their efficiency and reduce potential side effects when tested in young subjects (Wisniewski and Goni, 2015).

*In vivo* imaging of amyloid plaques is useful to evaluate anti-amyloid therapies and/or mechanisms associated with amyloid plaque production either at the clinical or preclinical levels. In humans, *in vivo* neuroimaging studies of amyloid plaques are performed with Positron emission tomography (PET) using different PET ligands (Nordberg, 2007). However, the low spatial resolution of PET does not allow the visualization of individual plaques. In animals, PET studies have provided controversial results (Klunk et al., 2005; Maeda et al., 2007) and, to date, PET has not been used to monitor anti-amyloid therapies. Other imaging modalities, such as optical imaging (Hintersteiner et al., 2005) or two-photon imaging after craniotomy (Dorostkar et al., 2014), have also been developed to detect amyloid plaques in animals. As PET, optical imaging does not detect individual plaques. On the contrary, two-photon imaging can reveal individual amyloid plaques at very high resolution (1 μm). It can detect plaques localized underneath a skull open window using non-destructive multiphoton laser excitation and images can be efficiently acquired from cortical surface up to 800 μm of depth. The field of view of the technique is limited and does not allow to record images from the whole brain as this would require large craniotomies (Delatour et al., 2010).

Continuous efforts are also ongoing to implement amyloid plaque detection by magnetic resonance imaging (MRI; Poduslo et al., 2002; Zaim Wadghiri et al., 2003; Higuchi et al., 2005; Sigurdsson et al., 2008). MRI-based monitoring of amyloid plaques can be divided into three research fields. Some methods are based on the natural contrast of the plaques that appear as dark spots in T2, T2^∗^-weighted (T2^∗^w; Jack et al., 2005; Dhenain et al., 2009) or susceptibility-weighted images (Chamberlain et al., 2009) due to the presence of iron in the core of these lesions. However, in humans the possibility to detect iron within plaques is still controversial (Dhenain et al., 2002; Meadowcroft et al., 2009; Zeineh et al., 2015). In addition, iron accumulation in mice mainly occurs in old animals, which makes amyloid plaque detection and pharmacology studies using this method very challenging in young animals. The use of MR contrast agents targeting amyloid plaques provides another option to detect these lesions. These agents modulate the MR signal of the plaques and increase their contrast with the brain parenchyma (Poduslo et al., 2002; Zaim Wadghiri et al., 2003; Higuchi et al., 2005; Sigurdsson et al., 2008). The third option to detect amyloid plaques by *in vivo* MRI is to use non-targeted contrast agents (Petiet et al., 2012). In that case, the non-targeted agents increase the signal of brain tissues that surround the plaques. As the volume of brain tissue is high, as compared to the volume of the plaques, these agents induce a high signal increase in the brain. This latter can be converted into resolution enhancement in order to record high resolution images.

The ability to use MR imaging to follow-up anti-amyloid therapies is still an opened question. Two studies showed that *ex vivo* MRI can be used to evaluate the impact of memantine or coenzyme Q10 on amyloid load (Scholtzova et al., 2008; Yang et al., 2011), but to our knowledge no study evaluated the ability of MRI to follow-up *in vivo* and in a longitudinal way the impact of anti-amyloid therapies on amyloid load. Here, we used Gd-stained MRI to monitor, in a longitudinal study, the efficacy of a passive immunotherapy (SAR255952).

SAR255952 is a mouse monoclonal aglycosylated IgG1 antibody engineered to reduce the risk of vasogenic edemas and microhemorrhages that have been associated to anti-amyloid immunotherapies (Pradier et al., 2013). These potential side effects of immunotherapies have been revealed by clinical trials that highlighted signal changes on MR images [also called amyloid imaging related abnormalities (ARIA); Sperling et al., 2011]. SAR255952 was designed on the basis of an antibody (13C3) that binds to soluble protofibrillar and fibrillar forms of Aβ (Schupf et al., 2008), the most synaptotoxic forms of Aβ (Haass and Selkoe, 2007). 13C3 do not target soluble Aβ monomers or low molecular weight Aβ complexes (Schupf et al., 2008), which limits peripheral sink effects that can lead to microhemorrhages (Pradier et al., 2013). SAR255952 is an aglycosyled form of 13C3. The rationale for this aglycosylation is based on the fact that classical anti-amyloid monoclonal glycosylated antibodies can induce Fc-γ receptor-mediated overactivation of microglial cells as well as activation of the complement pathway that may contribute to an inappropriate proinflammatory response leading to vasogenic edemas or cerebral microhemorrhages (Lue and Walker, 2002; Adolfsson et al., 2012). These effector functions of antibodies are linked to their glycosylated states (Jefferis, 2009). Aglycosylation limits the activation of Fc-γ receptors and C1q component of complement and preclude the risk of vasogenic edemas and microhemorrhages (Pradier et al., 2013). A phase 1 clinical trial is ongoing using a humanized version of this antibody^1^.

Transgenic mouse models of amyloidosis were treated between the age of 3.5 and 8.5 months and were imaged by MRI twice at 5.5 and 8.5 months. Gd-stained longitudinal MRI allowed to follow-up individual amyloid plaques from 5.5 to 8.5 months and revealed that plaques that were visible at the first time point were still detected at the second time point. Also, MRI, as well as histology, showed a reduction of amyloid load in the SAR255952-treated animals.

## Materials and Methods

### Animals

Experiments were conducted on female APP/PS1 (*n* = 26) and PS1 (*n* = 20) transgenic mice overexpressing amyloid precursor protein (APP) and/or presenilin 1 (PS1) mutations associated with familial AD [double Thy1 APP751_SL_ Swedish (KM670/671NL) and London (V717I) mutations introduced in the human APP751 sequence] × HMG PS1_M146L_ transgenic mouse line (Blanchard et al., 2003; Delatour et al., 2006). The PS1 mice were littermates of the APP/PS1 mice. In APP/PS1 mice, amyloid deposition starts at the age of 2.5 months (Blanchard et al., 2003). PS1 animals are amyloid free. Animal experimental procedures were performed in strict accordance with the recommendations of the EEC (86/609/EEC) and the French national committee (decree 87/848) for the care and use of laboratory animals. The research was conducted under the authorization number 91–326 from the “Direction Départementale des Services Vétérinaires de l’Essonne.”

### Passive Immunization

Animals were passively immunized weekly by intraperitoneal (IP) injections of a mouse monoclonal aglycosylated IgG antibody [SAR255952, 10 mg/kg (diluted in PBS)]. Age-matched control mice received weekly intraperitoneal injections of a control antibody that does not recognize murine antigens [DM4, 10 mg/kg (diluted in PBS), IP]. Four groups of mice were used: (1) APP/PS1 mice immunized with the SAR255952 antibody (*n* = 14); (2) APP/PS1 mice immunized with the control DM4 antibody (*n* = 12); (3) PS1 mice immunized with the SAR255952 antibody (*n* = 10) and (4) PS1 mice immunized with the control DM4 antibody (*n* = 10). All animals were 3.5-months-old at the beginning of the immunization and were treated for 5 months, i.e., until 8.5 months. The PS1 amyloid-free animals survived during the whole study while mortality was higher in the APP/PS1 mice (Supplementary Figure S1). All the mice were imaged by MRI at 2 and 5 months after the beginning of the treatment, i.e., at 5.5 and 8.5 months-old. We did not perform any baseline MRI (at 3.5 months) as we knew from previous experiments that amyloid plaques cannot be detected by MRI at this age. The mice were sacrificed after the second MRI exam.

### *In Vivo* Detection of Amyloid Plaques by MRI

Detection of amyloid plaques was based on the administration of a gadolinium derivative contrast agent, gadoterate meglumine (Gd-DOTA, Dotarem^®^, Guerbet, France), to the animals as previously described (Petiet et al., 2012; Santin et al., 2013). The contrast agent can be administered either intracerebro-ventricularly (Petiet et al., 2012) or intravenously combined with ultrasound-induced blood–brain barrier (BBB) opening (Santin et al., 2013). As BBB opening with ultrasound was recently shown to reduce amyloid load (Jordao et al., 2013), we opted for the intracerebro-ventricular administration of Gd rather than IV combined with ultrasound-induced BBB opening. Briefly, the mice were anesthetized with a mixture of isoflurane (1–2%) and air (1 L/min). They were then placed on a stereotaxic frame and the MR contrast agent was injected into the lateral ventricles at coordinates A/P -0.2 mm, L ± 1 mm, -1.8 mm relative to the surface of the dura mater (Paxinos and Franklin, 2001) by using Hamilton syringes (26 s gauge). A volume of 1 μl (0.5 mmol/mL, i.e., 0.02 mmol/kg) was injected into each hemisphere at a rate of 0.2 μl/min. *In vivo* MRI was performed on a 7T-spectrometer (Agilent, USA) interfaced with a console running VnmrJ 2.3. The spectrometer was equipped with a rodent gradient insert of 700 mT/m. A birdcage coil (RapidBiomed, GmbH, Germany) was used for emission and an actively decoupled mouse brain surface coil (RapidBiomed GmbH, Germany) was used for reception. MR images were recorded using a high-resolution 3D-Gradient Echo sequence with a resolution of 29^∗^29^∗^117 μm^3^ (field of view: 15^∗^15^∗^15 mm^3^, matrix = 512^∗^512^∗^128, TR = 50 ms, TE = 25 ms, flip angle = 20°, number of averages = 2, bandwidth = 25 kHz, acquisition time: 1 h 49 min). MR images were recorded starting at 60 min after administration of the Gd-DOTA contrast agent used for Gd-staining. During the MRI experiment the animals were anesthetized with a mixture of isoflurane (0.75–1.5%) and carbogen (95% O_2_ – 5% CO_2_) and their breathing rate was monitored. Carbogen was used to reduce the signal coming from the circulating blood (Thomas et al., 2003).

### Amyloid Load Quantification from MR Sections

**FIGURE 1:**
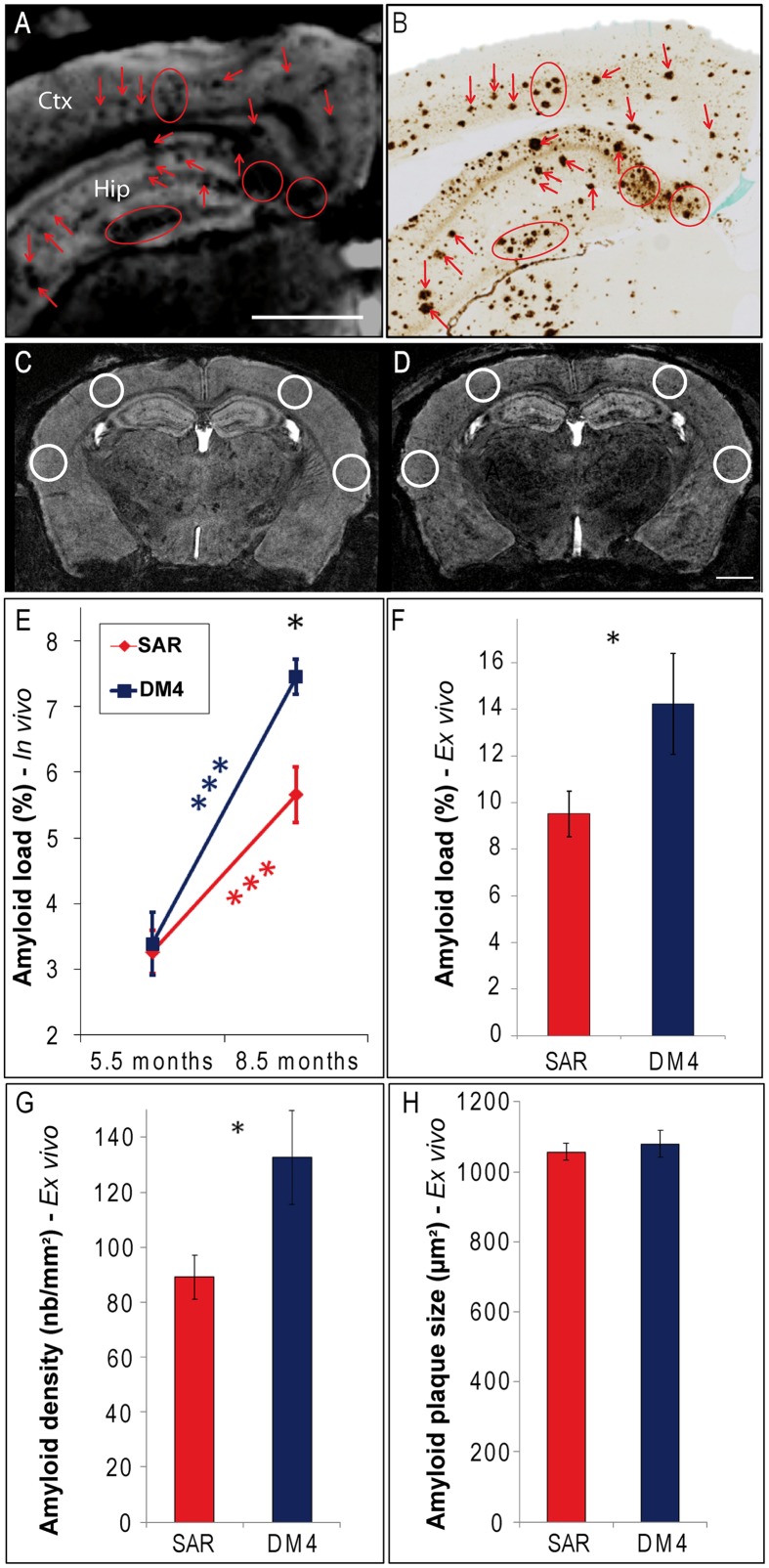
**Modulation of amyloid load following immunotherapy with SAR255952 or DM4 monoclonal antibodies**. Registration between MR images **(A)** and amyloid-stained histological sections (**B**; 6E10 immunohistochemistry) in an 8.5-months-old APP/PS1 mouse. Hypointense spots observed on the MR images at the level of the cortex (Ctx) and hippocampus (Hip; **A**) are localized with amyloid plaques detected on the histological sections **(B)**. ROIs used for plaque counting displayed on MR images of an APP/PS1 mouse at the age of 5.5 **(C)** and 8.5 months [**D**; A/P level = -2.2 mm compared to the Bregma (Paxinos and Franklin, 2001)]. **(E)** Measures from MR sections revealed an increased amyloid load between 5.5 and 8.5 months in SAR255952 and DM4-treated animals (repeated measure ANOVA and *post hoc* analysis within each group *F*[1,11] = 23 and 29, respectively, ^∗∗∗^*p* < 0.001). At 8.5 months, the amyloid load was lower in SAR255952-treated animals (*n* = 9) compared to control DM4-treated mice (*n* = 4; repeated measure ANOVA and *post hoc* analysis *F*[1,11] = 7, ^∗^*p* = 0.02). Histological measures confirmed the lower amyloid load [*F*, Student’s *t*-test, *t*(10) = 2.3, ^∗^*p* = 0.04] and reduced density of amyloid plaques [**G**, *t*(10) = 2.7, ^∗^*p* = 0.02] in SAR255952-treated animals at 8.5 months. The average size of the amyloid plaques was not modulated by therapy [**H**, *t*(10) = 0.6, ns]. Amyloid load in **(E,F)** is expressed as the proportion (%) of tissue area occupied by hypointense spots **(E)** or 6E10 immunoreactivity **(F)**. Scale bars: 1 mm. Error bars represent standard error of the mean.

Magnetic resonance images were filtered with a kernel defined in matrix form as 1 1 1; 1 8 1; 1 1 1 with ImageJ freeware (Schindelin et al., 2012). Then cortical amyloid load was calculated by using a method similar to that reported by Jack et al. (2005) and Santin et al. (2013). Briefly, eight coronal slices (antero-posterior direction), evenly spaced by 468 μm, were selected and four circular ROIs (surface ∼1 mm^2^ each) were drawn on each of these slices (two in each hemisphere; ImageJ freeware, **Figures 1C,D**). The eight slices were positioned so that the third slice was localized at the level of the anterior commissure. The coordinates of the first and last slices were thus 1.08 and -2.2 mm compared to the Bregma (Paxinos and Franklin, 2001). Hypointense spots were manually outlined, excluding hypointense elements that could be followed over more than two adjacent slices, or that had a tube-like shape, suggesting the presence of a blood vessel. Plaque load corresponded to the ratio of the mean area of hypointense spots measured in each ROI over the surface of the ROI.

### Neuropathology

After the last MRI, the animals received an injection of pentobarbital. They were then perfused by intracardiac injection with PBS 0.01 M SIGMA + Heparin 5000 units/L and 10% neutral buffered formalin (PB 0.1 M) using a peristaltic pump (flow rate ∼2 mL/min). The brains were then carefully extracted, post-fixed in formalin for 24-h at 4°C and finally stored in PBS at 4°C.

The brains were then processed by NeuroScience Associates^2^ in order to perform histological analysis. Formalin-fixed brains were frozen and cut along the rostro-caudal axis. Five series of 25-μm-thick coronal sections were collected. The first, second and third series were used for Nissl staining, Aβ peptide deposits staining (6E10 monoclonal antibody IHC), and immunoglobulin G (IgG) staining (antimouse secondary antibodies IHC), respectively. A flatbed scanner (ImageScanner III, G.E. Healthcare) was used to digitize all stained series (lateral resolution: 5 μm for sections Aβ- and IgG-stained sections). Thus, for each stain, 80 sections, evenly spaced by 125 μm across the entire cerebrum were digitized.

Amyloid plaques and IgG staining were quantified from histological sections by using ImageJ and a protocol similar to that used to quantify plaques from MRI: eight coronal slices stained with 6E10 or anti-IgG, evenly spaced by 500 μm, were selected and four circular ROIs (surface ∼1 mm^2^ each) were drawn on each of these slices (two in each hemisphere). Each stained section was binarized after conversion of the images into gray scale images and thresholded. Amyloid plaques were further segmented by using a watershed algorithm. The proportion of tissue area occupied by 6E10 or anti-IgG immunoreactivity, as well as the number of amyloid plaques and their average sizes were quantified within each ROI by using the “area fraction” and “analyze particles” tools of the imageJ measurement process. The ratio (stained surfaces/ROI surfaces) was taken as a global measure of the cerebral “amyloid load” or “IgG load” of each individual mouse. Thus, for each staining, 64 observations were performed for each animal.

### Correlation between *In Vivo* MRI and Histology

Longitudinal MR images and histological sections for a given animal were registered by using BrainVisa freeware^3^ (Dubois et al., 2010). A rigid registration was performed to register MR images recorded at 8.5 months onto MR images recorded at 5.5 months.

### Statistical Analysis

Statistical analysis was performed using Statistica 7 software (Statsoft, France). The significance of between-group differences was tested by repeated measures ANOVA for longitudinal study and Student’s *t*-test for *post mortem* measures. Correlation studies were based on Pearson’s correlation coefficient. Statistical significance was set to *p* < 0.05. Results are expressed in figures as mean ± standard error of the mean (SEM).

## Results

### Detection of Amyloid Plaques by Gd-Stained MRI

**FIGURE 2:**
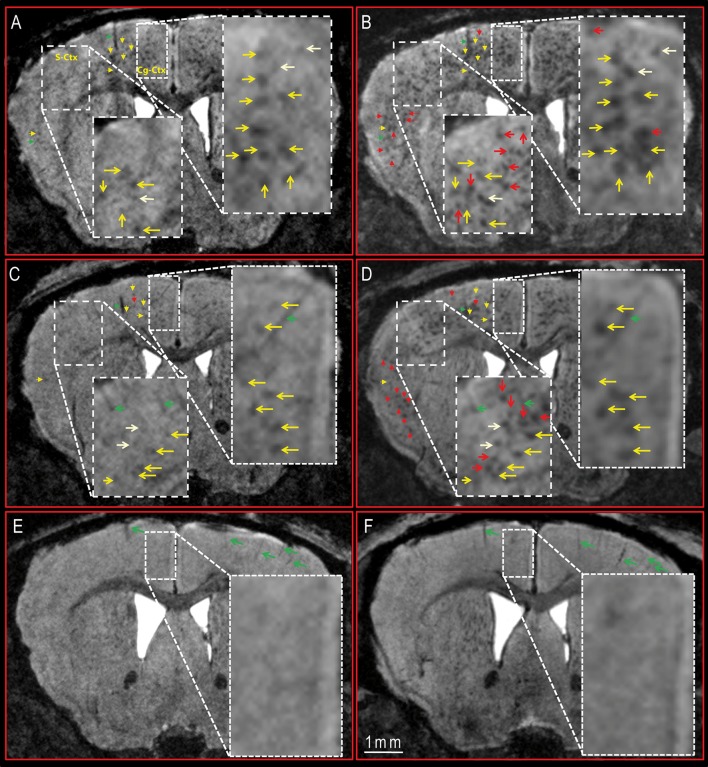
**Longitudinal follow-up of DM4 and SAR255952 treatments by MR imaging in APP/PS1 and PS1 mice.** DM4 **(A,B)** and SAR255952-treated **(C,D)** APP/PS1 mice and DM4-treated PS1 amyloid-free mice **(E,F)** were imaged by MRI at the age of 5.5 **(A,C,E)** and 8.5 months **(B,D,F)**. Hypointense spots corresponding to amyloid plaques were visible in the APP/PS1 mice **(A–D)**, but not in the PS1 animals **(E,F)**. MR images from the same animals were registered to follow-up these hypointense spots between 5.5 and 8.5 months. In both DM4 **(A,B)** and SAR255952-treated **(C,D)** APP/PS1 mice the plaques detected at 5.5 months were still visible at 8.5 months (yellow arrows). Some new plaques occurred between the age of 5.5 and 8.5 months (red arrows). No plaques were detected in PS1 mice at 5.5 or 8.5 months. Typical landmarks, such as blood vessels are shown with green arrows. Some plaques that were slightly visible at 5.5 months and which were more visible at 8.5 months are labeled with pale yellow arrows. S-Ctx, somatosensory cortex; Cg-Ctx, cingulate cortex.

Three-dimensional MR images of APP/PS1 and PS1 mice were recorded with a resolution of 29^∗^29^∗^117 μm^3^. Hypointense spots were seen in the cortex (**Figures 1A** and **2A–D**), hippocampus (**Figure 1A**), thalamus and septal nuclei of transgenic APP/PS1 mice after Gd-staining protocols. No hypointense spots were observed in the brain of PS1 amyloid-free mice at 5.5 and 8.5 months (**Figures 2E,F**). Registration between MR images and histological sections confirmed that the hypointense spots on MR images were amyloid plaques (**Figures 1A,B**).

### Longitudinal Monitoring of Amyloid Plaques and Treatment Effects

APP/PS1 mouse models of amyloidosis were passively immunized with SAR255952 or with a control (DM4) antibody from 3.5 months old until 8.5 months-old. The mice were followed-up in a longitudinal way and MR images were recorded 2 and 5 months after the beginning of the treatment, i.e., at 5.5 and 8.5 months-old. For each animal, we registered MR images recorded at 5.5 and 8.5 months in order to monitor the evolution of each hypointense spots corresponding to amyloid plaques. The visual observation of these images revealed an age-related increased in amyloid load in both DM4 (**Figures 2A,B**) and SAR255952-treated animals (**Figures 2C,D**). In both treatment groups the plaques that were present in 5.5-months-old animals were still present at 8.5 months (**Figures 2A–D** – yellow arrows). Some new plaques occurred between the age of 5.5 and 8.5 months (**Figures 2A–D** – red arrows).

Quantitative studies of MRI sections revealed a similar amyloid load in the 5.5-months-old animals treated with DM4 or SAR255952 antibodies (n.s., **Figure 1E**). Amyloid load increased in both DM4 and SAR255952-treated animals between the ages of 5.5–8.5 months (from 3.4 ± 0.6% to 7.5 ± 0.2% and from 3.3 ± 0.3% to 5.7 ± 0.4% in DM4 and SAR255952-treated mice, respectively, *p* < 0.001, **Figure 1E**). At 8.5 months, the amyloid load detected by MRI was 24% lower in the SAR255952-treated mice compared to the DM4-treated mice (*p* = 0.02, **Figure 1E**). Histological evaluations confirmed the lower amyloid load in 8.5-months-old APP/PS1 animals treated with SAR255952 compared to animals treated with the control antibody (15.8 ± 2.0% versus 10.6 ± 1.1%, respectively, *p* = 0.04, **Figure 1F**). In addition, histological quantifications revealed that the number of amyloid plaques (plaque density) was reduced by 33% in the SAR255952-treated animals compared to the DM4-treated animals (*p* = 0.02, **Figure 1G**), whereas the size of the plaques was not significantly different in the two groups (**Figure 1H**).

### Relationship with Immunoglobulin and Amyloid Load in the Brain

**FIGURE 3:**
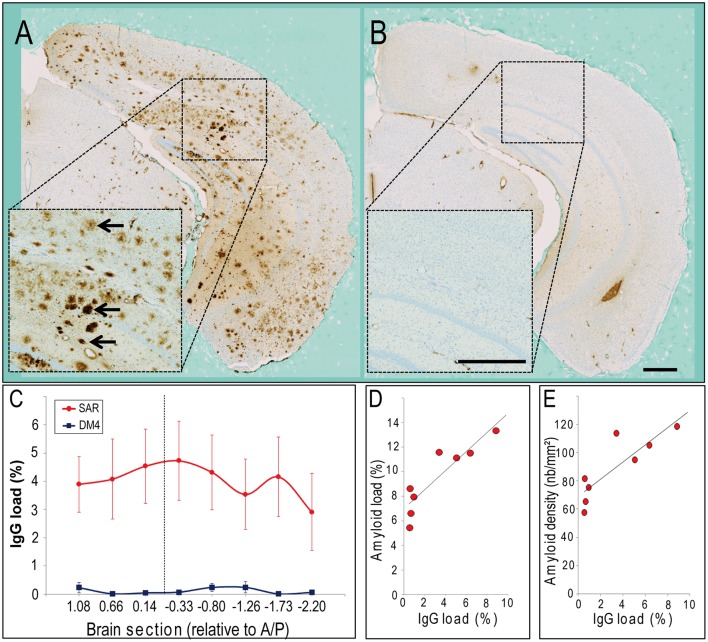
**Evaluation of IgG staining in the brains of APP/PS1 mice treated with SAR255952 or DM4**. IgG staining in the brains of APP/PS1 mice treated with SAR255952 **(A)** or DM4 **(B)**. IgG was only decorating amyloid plaques (black arrows) in the SAR255952-treated animals **(A)**. **(C)** IgG load in the different brain sections. The site of contrast agent administration (A/P -0.2 mm) is highlighted with a dashed line. IgG load was higher in SAR255952 compared to DM4-treated animals. The IgG load was similar within all brain sections in SAR255952 (*n* = 9) or DM4-treated (*n* = 4) animals. **(D,E)** Relationship between IgG and amyloid load (**D**, *r* = 0.90, *p* = 0.002) or density of amyloid load (**E**, *r* = 0.85, *p* = 0.007) quantified from histological sections. IgG and amyloid loads in **(C–E)** are expressed as the proportion (%) of tissue area occupied by anti-IgG or 6E10 immunoreactivity. Error bars stand for standard error of the mean. Scale bars: 500 μm.

We stained IgG deposition in the brain to further evaluate the interaction between IgG and amyloid. In SAR255952-treated animals, IgG decorated amyloid plaques from all the brain regions examined (**Figures 3A,C**) while we did not detect any plaques labeled by IgG in the DM4-treated animals (**Figures 3B,C**). The quantification of IgG in brain sections spreading from the anterior to the posterior parts of the brain showed that IgG was equally distributed on all the brain sections (**Figure 3C**). There was also a strong positive correlation between the IgG load and the amyloid load (*r* = 0.90, *p* = 0.002, **Figure 3D**) or amyloid density (*r* = 0.85, *p* = 0.007, **Figure 3E**) detected by histology.

## Discussion

The formation of amyloid plaques is one of the major neuropathological hallmarks of AD. Limiting the amyloid load in the brain is therefore a potential therapeutic strategy against this disease. Anti-amyloid immunotherapies are the approaches that have come furthest to limit cerebral amyloidosis. Current refinements of these therapies aim to improve their efficiency and reduce potential side effects. Methods of *in vivo* amyloid plaques detection are drug development tools that are critical to speed the development of these new therapies (U.S. department of health and human services et al., 2014). PET (Nordberg, 2007) or optical imaging (Hintersteiner et al., 2005) can be used to quantify amyloid load but these methods cannot detect individual plaques. On the contrary, several MRI approaches have been developed to detect individual amyloid plaques, but none of them were used to follow-up a therapy *in vivo* and in a longitudinal way. Some methods are based on the natural contrast of the plaques (Jack et al., 2005; Chamberlain et al., 2009). Some others use targeted MR contrast agents that modulate the MR signal of the plaques and increase the contrast between the plaques and the brain parenchyma (Poduslo et al., 2002; Zaim Wadghiri et al., 2003; Higuchi et al., 2005; Sigurdsson et al., 2008). Here, we used a third method, called Gd-staining, based on non-targeted contrast agents. One of the advantages of the Gd-staining protocol is that it allows to record images with a much better resolution and contrast than without or with targeted contrast agents, as it is based on the distribution of the agents within the brain parenchyma and not into the plaques. We showed that Gd-stained MRI is able to detect amyloid plaques *in vivo* with a very high resolution (29 μm × 29 μm × 117 μm). It can be used to monitor individual amyloid plaques in the whole brain and to assess the dynamics of their formation and clearance. Interestingly, we were able to demonstrate the very long stability (3 months) of plaques already deposited in the brain. One can argue that the registration of amyloid plaques detected from Gd-stained MRI and immunohistochemical sections was not perfect. This might be related to limitations in the precision of co-registration because of the different thickness of immunohistochemical (25 μm) and MRI sections (117 μm). This can also be related to the different mechanisms leading to plaque detection by MRI or immunohistochemistry. With Gd-stained MRI, the ability to detect the plaques is related to their hydrophobicity (Dhenain et al., 2006), while by immunohistochemistry it is associated to the presence of amyloid epitopes revealed with specific antibodies.

It is important to outline that the doses of Gd administered to the animals were very low (0.02 mmol/kg) as compared to doses commonly used for intra-venous administration (0.1–0.2 mmol/kg). Also, the contrast agent that we used is largely used in the clinic including for applications that are based on its penetration into the brain (Anzalone et al., 2013; Saake et al., 2016). Its safety profile is supported by post-marketing experience from approximately 30 million doses administered in 70 different countries since its initial approval in 1989 (Guerbet, 2013). Finally, a recent study showed that unlike linear gadolinium-based contrast agents macrocyclic contrast agent such as Dotarem does not accumulate in the brain even after 20 intra-venous administration at high doses (0.6 mmol/kg; Radbruch et al., 2015; Robert et al., 2016). The only limitation of the Gd-staining method is that it is based on the intracerebro-ventricular administration of the contrast agent, although this administration way is widely used in preclinical settings. Albeit still speculative, several procedures can be proposed to by-pass the BBB and promote future development of Gd-staining procedure including in humans. Gadolinium can be administered into the subarachnoid space and reach the brain as already demonstrated in human studies (Tali et al., 2002; Selcuk et al., 2010). Gadolinium can also be administered intravenously in association with BBB opening based on ultrasound technology (Santin et al., 2013). Interestingly, this method of BBB opening in currently tested in humans^4^. Compounds that transiently increase BBB permeability, such as lysophosphatidic acid (LPA), could also be administered together with gadolinium to increase its uptake into the brain (On et al., 2013). Finally, methods based on natural transport systems could also be used to improve the uptake of gadolinium across the BBB. For example, a recent study showed that fusing a single-chain Fab fragment of an anti-transferrin receptor antibody to a molecule of interest can increase the uptake of the molecule of interest by more than 50-fold (Niewoehner et al., 2014).

Multiple mechanisms have been involved in the efficacy of anti-amyloid immunotherapies. For example, some of them clear aggregated forms of Aβ (Demattos et al., 2012), whereas others focus on inhibiting amyloid seeding to prevent amyloid deposition propagation (Golde, 2003) or target soluble forms of Aβ to shift the equilibrium between soluble and fibrillar Aβ to favor plaque dissolution (DeMattos et al., 2001). The SAR255952 anti-amyloid antibody used in this study targets both protofibrillar and fibrillar amyloid forms (Schupf et al., 2008; Pradier et al., 2013). Our data show that IgG (presumably SAR255952) is homogeneously distributed in the brain of SAR255952-treated APP/PS1 mice while DM4-treated APP/PS1 mice did not display any IgG immunoreactivity. We also found a positive relationship between the amyloid and IgG loads in the SAR255952-treated APP/PS1 mice which is consistent with the targeting of fibrillar amyloid forms of Aβ by SAR255952. By using Gd-stained MRI, we were able to demonstrate the efficacy of this anti-Aβ antibody to slow down the evolution of amyloid load. We could also monitor individual plaques during two imaging sessions separated by 3 months and showed that, even in the SAR255952-treated animals, plaques that were present at the first time point were still detected 3 months later. This suggests that the targeting of fibrillar forms of Aβ by SAR255952 does not lead to the clearance of the plaques. The lower amyloid load in the SAR255952-treated animals was thus due to a decrease in the formation of new plaques, probably related to the targeting of protofibrillar forms of Aβ.

On the basis of our histological study, we also reported that the lower amyloid load in the SAR255952-treated animals was linked to a reduction of amyloid plaque density and not to a diminution of the size of the plaques. One explanation for this pattern of amyloid load modulation can come from recent models showing that, in the absence of therapy, amyloid load increases following three temporal phases (Burgold et al., 2014). In the first phase, the density of plaques increases while the size of the plaques is stable. In the second phase, the size of the plaques increases while their density remains stable. Plaque size and volume are stable in the last phase. The modulation of the density of the plaques and not of their size in our animals is consistent with an action of the therapy in mice that were in the first phase of amyloid load increase, i.e., in the phase where it is the density of the plaques and not their size that evolves over time and indeed, the relatively young animals involved in our study were probably in that stage of plaque development.

## Conclusion

We have shown that Gd-stained MR imaging can be used to monitor individual amyloid plaques in a longitudinal way, to test the efficacy of an anti-amyloid immunotherapy in APP/PS1 mice. This method can be used in addition to histological evaluations as a useful tool for the development of new therapies. Future improvement of the method may be based on the automatic segmentation of the amyloid load to speed-up plaque quantification after Gd-staning (Iordanescu et al., 2012). Also, our study showed that individual plaque labeling is feasible *in vivo* with a conventional MR contrast agent, as long as the contrast agent remains furtive for the BBB.

## Author Contributions

MS, Thomas Debeir, TR, and MD designed the study. LP, CC, Thomas Debeir, TR provided the immunotherapy. Thomas Debeir, TR, and MD coordinated the study. MS and MD performed the MRI experiments and analysis. MS, MV, A-SH, Thierry Delzescaux, and MD designed and performed the histological analysis. MS and MD wrote the manuscript. MV, A-SH, Thomas Debeir, and TR revised the manuscript.

## Conflict of Interest Statement

LP, CC, TD, and TR are full employees by Sanofi. All other authors declare that the research was conducted in the absence of any commercial or financial relationships that could be construed as a potential conflict of interest.

## Footnotes

1 http://www.alzforum.org/therapeutics/sar228810.

2 www.neuroscienceassociates.com

3 http://brainvisa.info

4 https://clinicaltrials.gov/ct2/show/NCT02343991.
