# Supplementary material for: In Vivo Detection of Amyloid Plaques by Gadolinium-Stained MRI Can Be Used to Demonstrate the Efficacy of an Anti-amyloid Immunotherapy
Source: Front Aging Neurosci. 2016 Mar 22;8:55. doi: 10.3389/fnagi.2016.00055 (PMC4802995; doi:10.3389/fnagi.2016.00055)

Supplementary Figure 1. Animal survival during the immunization trial

Kaplan-Meir curves showing the survival distribution function in APP/PS1 (full lines) and PS1 (dotted lines) mice treated with SAR255952 (red) and control DM4 (blue) antibodies. Squares and circles indicate animal death. * highlights a higher survival rate in APP/PS1 animals treated with SAR255952 compared with animals treated with DM4 antibodies (67 versus 33%, Cox's F-test, $F(10, 16) = 2.4$, $p=0.05$). The death of the animals occurred regularly during the whole study period and could not be easily associated to any symptom or particular event.The labels "MRI 1" and "MRI 2" indicate the timing of the MR acquisitions.

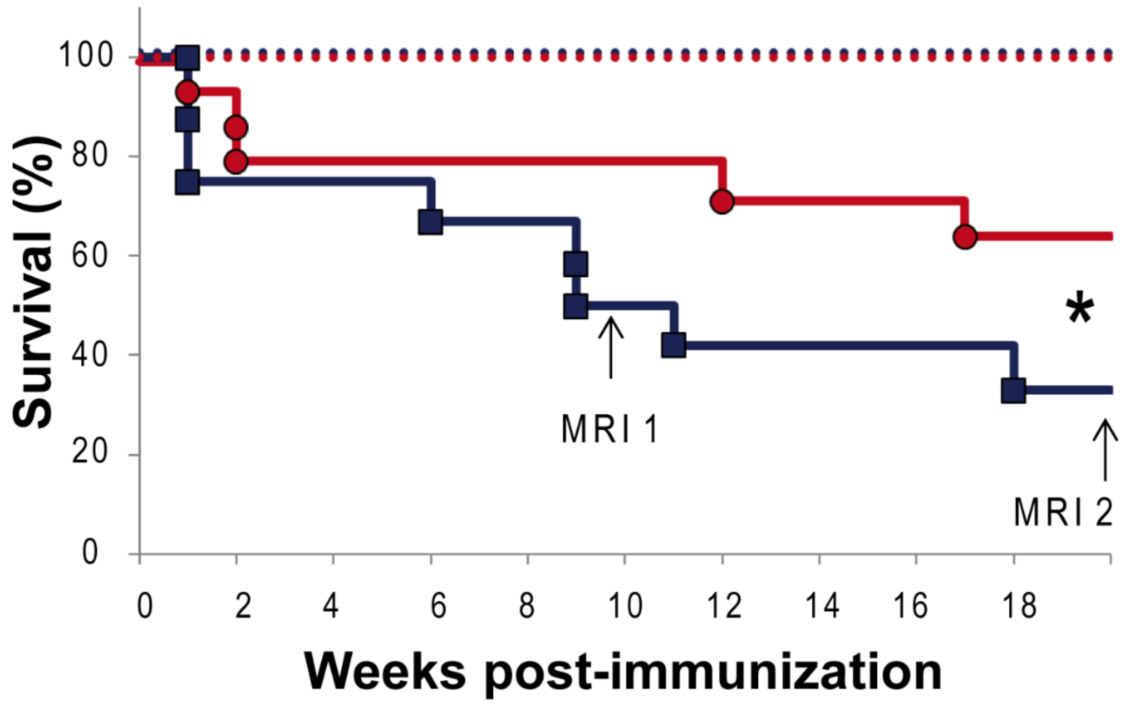

Supplement: Supplementary file 1 [file Image_1.PDF]
